# Electro-acupuncture for protracted amphetamine abstinence syndrome: study protocol for a pragmatic randomized controlled trial

**DOI:** 10.1186/s13063-022-06154-7

**Published:** 2022-03-15

**Authors:** Huan Ren, Yiwei Zeng, Min Zhang, Su Zhang, Zhihan Chen, Binbin Wu, Jun Liu, Yulan Ren

**Affiliations:** 1 School of Acupuncture-Moxibustion and Tuina, Chengdu University of Traditional Chinese Medicine, Chengdu, Sichuan 610075 China; 2 Coercive Rehabilitation Center for Addicts Affiliated to the Public Security Bureau of Deyang, Deyang, Sichuan 618007 China; 3 Medical Rehabilitation Department, Drug Rehabilitation Administration of Sichuan Province, Chengdu, Sichuan 610036 China; 4 School of Chinese Classics, Chengdu University of Traditional Chinese Medicine, Chengdu, Sichuan 610075 China

**Keywords:** Electro-acupuncture, Protracted amphetamine abstinence syndrome, Pragmatic randomized controlled trials (pRCTs), Clinical trial protocol

## Abstract

**Background:**

Protracted amphetamine abstinence syndrome is one of the primary causes of relapse for amphetamine-type drug abusers during withdrawal. However, the importance of the management of protracted amphetamine abstinence syndrome is underestimated. Electro-acupuncture may be a safe and effective alternative therapy for protracted amphetamine abstinence syndrome, but the evidence is limited.

**Methods:**

The study is a prospective, two-center, randomized, waitlist controlled, open-label pragmatic trial. A total of 300 patients with protracted amphetamine abstinence syndrome will be recruited. All participants will be randomly assigned to an electro-acupuncture group or a waitlist group in a 1:1 ratio. Participants in the electro-acupuncture group will receive the electrical-acupuncture treatment. Waitlist group participants will not receive electro-acupuncture treatment but will be assessed at each visit. Treatments will be administered twice a week for a total of 4 consecutive weeks. The primary outcome in this study is the change in the ACSA between baseline (week 0) and the completion of treatment (week 4), and the secondary outcomes are changes in the Hamilton Depression Scale (HAMD), the visual analog scale (VAS), the Hamilton Anxiety Scale (HAMA), the Pittsburgh Sleep Quality Index (PSQI), the Montreal Cognitive Assessment (MoCA), and the Medical Outcomes Study 36-item Short-Form Health Survey (SF-36).

**Discussion:**

This study will assess the effectiveness of acupuncture in PAAS in real-world settings to provide support for clinical decisions and a basis for subsequent trials comparing acupuncture with other positive regimens.

**Trial registration:**

ClinicalTrials.gov ChiCTR2000040619. Registered on 3 December 2020

## Background

Amphetamine-type stimulants (ATS) are one of the most widely abused psychostimulative substances and are of highly addictive potential. Over the past few years, the increasing trend in ATS abuse has become a major public health problem around the globe. In 2019, around 27 million people worldwide, accounting for 0.5% of the global adult population, have used ATS [1], and more than one-third of these 27 million users was in East and South-East Asia [2]. The acute effects of ATS administration mainly include hallucination, sexual disinhibition, and irritation [3, 4], while chronic administration could cause cognitive impairments, social dysfunction, and multiple neuropsychiatric complications [5, 6]. There are also reports of severe consequences by overdose (e.g., hyperthermia, myocardial infarction, insomnia, and renal failure) [7]. Some ATS abusers will have to suffer long-lasting physical and mental symptoms such as body discomfort, depression, anxiety, and increasing drug craving even after a long time of withdrawal, which is defined as the “Protracted amphetamine abstinence syndrome (PAAS)” [8, 9]. The persistence of PAAS exerts an adverse impact on patients after ATS withdrawal, hinders them from backing to normal life, and probably accounts for relapse, which makes the clinical management of PAAS quite crucial.

However, there are still no medications available for the treatment with ATS use disorder, not to mention the PAAS [10], and cognitive behavioral therapy (CBT) and symptomatic treatment are the main approaches [11, 12]. However, CBT requires a great deal of infrastructure support for treatment providers, and transportation to the treatment site may also be difficult for some patients. Using therapists trained in CBT may be difficult for many programs [13]. Clinically, antipsychotics are commonly used in the treatment of ATS addicts, while typical antipsychotics have many adverse reactions, such as extrapyramidal symptoms and sedation [14].

Acupuncture is an important component of traditional Chinese medicine (TCM); currently, increasing studies showed that acupuncture could alleviate symptoms related to substance use disorders [15, 16], suggesting that it may offer a promising non-pharmacological approach for protracted abstinence symptoms, especially electro-acupuncture (EA). A comparative study showed that electro-acupuncture could alleviate withdrawal syndrome, anxiety, and depression in patients with methamphetamine addiction, with no side effects, no dependence, and no drug interaction [17]. A randomized controlled trial (RCT) published recently reported that compared with sham acupuncture, EA could effectively attenuate the symptoms of psychosis, anxiety, and depression during abstinence in methamphetamine addicts [18]. Though these studies yielded robust evidence of the efficacy of EA treatment for ATS addiction, especially for the protracted withdrawal syndromes, the RCT design with small sample size and strict inclusion criteria might limit the generalizability of the evidence.

Pragmatic randomized controlled trials (pRCTs) are randomized controlled design for comparing the results of different interventions in clinical practice in real or near-real medical environments [19]. pRCTs emphasize practical applicability and generalizability in real-world settings (external validity) and usually focus on the overall effectiveness rather than on the efficacy of the intervention [20]. Moreover, it allows complex intervention and takes into account the treatment will of patients, which makes it more feasible and suitable using the pRCT design to evaluate the clinical effects of acupuncture therapy [21]. Therefore, we propose a two-center, randomized, waitlist-controlled pragmatic trial to critically investigate the effectiveness of acupuncture in the treatment of patients with PAAS to provide a new direction for clinical management of ATS use disorder.

## Materials and design

### Objectives

The aim of this study is to assess the effectiveness of electro-acupuncture for patients with PAAS in clinical practice.

### Hypothesis

We hypothesize that eight sessions of EA treatment over 4 weeks will result in alleviation of protracted abstinence symptoms in patients with PAAS.

### Study design

This protocol also follows the updated CONSORT Statement (www.consort-statement.org), the Standard Protocol Items: Recommendations for Interventional Trials (SPIRIT) Checklist, and the standards for reporting interventions in clinical trials of acupuncture (STRICTA) guidelines [22–24].

**Fig. 1:**
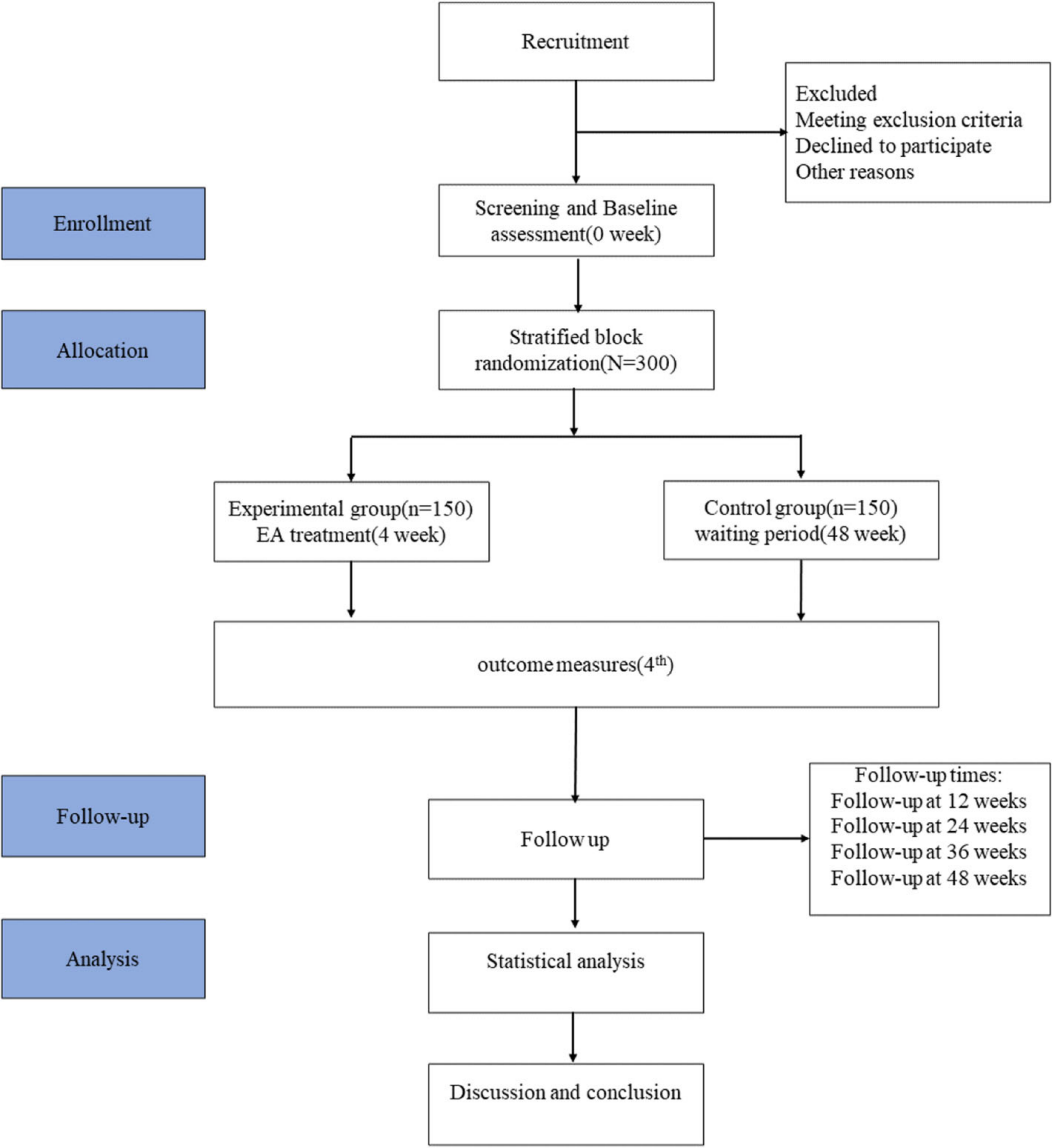
Trial procedure flow chart

A two-center, randomized, waitlist controlled, open-label pragmatic trial will be conducted, and a total of 300 patients are expected to be recruited from the Coercive Rehabilitation Center for Addicts Affiliated to the Public Security Bureau of Deyang City (Deyang 618000, P.R. China) and the Coercive Rehabilitation Center for Addicts Affiliated to the Public Security Bureau of Neijiang City (Neijiang 641000, P.R. China). Before the trial, all researchers will be given unified training. Placebo, sham acupuncture, and other placebo measures are generally not used as controls in pRCTs. A blank group can be adopted as a control if allowed by ethical principles, but the trial has to be conducted in a real-world setting [25]. The estimated duration of the main study phase is planned to last approximately 1 year. The flowchart of this study is shown in Fig. 1.

### Ethical approval

This study has been registered in the Chinese Clinical Trial Registry (identifier: ChiCTR2000040619, December 03, 2020.). The study protocol and informed consent (version no. 20210331, V3.0) have been systematically reviewed and approved by the Medical Ethics Committee of the Affiliated Hospital of the Chengdu University of Traditional Chinese Medicine (Approval No. 2019KL-067, September 21, 2020). All participants will voluntarily sign the informed consent form that has been approved by the ethics committee. Any important protocol modifications will be sent to the relevant parties through email.

### Participant recruitment and informed consent processes

The administrative staff of the Coercive Rehabilitation Center will be involved in the organization and supervision during the whole study process. Patients will be identified by a numerical code and no personal information will be included in the case report form (CRF) and the database or will be included in the publication. A detailed explanation of the study process will be provided by a trained researcher to the participants in a private room, which mainly includes the objective, methods, privacy security, possible risks, and participant rights. All participants will be informed that they can withdraw from the study at any time with the quit reasons recorded. All participants will be obtained their written informed consent prior to enrollment and kept one of the copies.

### Inclusion criteria

This study is designed as a pRCT based on a real-world setting. Broad inclusion and exclusion criteria are prearranged [25].

1. Patients aged from 18 to 60 who meet the diagnostic criteria in the Diagnostic and Statistical Manual of Mental Disorders, Fifth Edition (DSM-5) [26]
2. Negative in a urine test for ATS
3. Without mental disorders or any other severe organic diseases
4. Uninvolved in other clinical trials within 3 months
5. Signed the informed consent form

### Exclusion criteria

Patients with any of the following conditions will not be included in this study:

1. Local trauma or infection around the acupoints
2. Intolerance with EA and EA treatment or have a needle phobia
3. Pregnant or breastfeeding women

### Stopping and withdrawal criteria

If the physician and the principal investigator assert that there are risks of serious adverse events during the study, it will be stopped. During the research, participation is completely voluntary, and participants have the right to withdraw from the trial for any reason at any time.

### Randomization

All participants will be assigned, in a 1:1 ratio, to the EA treatment group and waitlist group using stratified block randomization. The randomization sequence will be generated using a computer-based random-digit generator before the recruitment and will be blinded to investigators. Randomization information will be sealed in an envelope and will be unsealed by the corresponding participant after recruitment. The randomization sequence and envelopes will be stored by an associated team that will not be involved in other parts of this study.

### Blinding

Given that this study is a waitlist-controlled design, both the participants and acupuncturists cannot be blinded. All outcome measures will be conducted by a third party who does not participate in the trial. Statistical analysis will be performed by a statistical expert uninvolved in research and evaluation.

### Interventions

#### EA treatment group

Acupuncture will be performed by licensed acupuncturists who have more than 3 years of acupuncture clinical experience. The acupuncturists will be trained on the use of the semi-standardized protocol, reporting adverse events (AEs), and other experimental procedures and will only be involved in the acupuncture treatment.

The acupoint selection regimen was developed based on traditional acupuncture theories and our previous data mining for relevant articles [27]. The standardized section is composed of bilateral acupoint stimulation to Shenmen (HT7), Neiguan (PC6), Zusanli (ST36), and Sanyinjiao (SP6). Furthermore, the acupuncturist will be allowed to treat no more than three additional acupuncture points according to the individual conditions. The locations of the acupoints conform to the standard defined by the World Health Organization [28].

Disposable sterilized needles made of stainless steel (0.25 × 40 mm and 0.25 × 13 mm purchased from Huatuo, Suzhou Medical Supplies Factory Co. Ltd, Suzhou, China) will be applied in this trial. Acupuncture needles will be inserted to a certain depth to achieve the De Qi sensation. De Qi is indicative of effectiveness during acupuncture treatment and is characterized by dull pain, heaviness, aching, tingling, and numbness. After the achievement of De Qi, the SDZ-III EA apparatus (Huatuo; Suzhou, China) will be used for bilateral electrical stimulation at SP6 and ST36. The EA apparatus will be set to generate sparse and dense waves with frequencies of 2 Hz and 100 Hz, respectively, and a current of about 10–15 mA.

The treatment for the EA group will consist of 8 sessions of EA treatment with a duration of 30 min per session administered over a period of 4 weeks (2 sessions per week). Follow-up will last for 48 weeks after the treatment period with 4 visits to collect adequate relief data.

#### Waitlist group

The waitlist group patients will receive no treatments during the trial period. After 48 weeks of follow-up, they then will receive EA treatment the same as the EA group.

### Concomitant treatments

All participants could receive any other treatment during the trial. All treatments will be recorded on the CRF.

### Outcome measures

#### Primary outcome measures

Amphetamine Cessation Symptom Assessment (ACSA) is a clinician-based scale to assess withdrawal symptoms of patients with ATS addiction, which includes three subscales of “anxiety and mood,” “fatigue,” and “craving.” Items are scored on a 5-point scale ranging from 0 to 4 with higher scores representing more severe PAAS. The ACSA has been validated to be of high sensitivity, reliability, and specificity [29].

The change in ACSA scores will be measured at baseline, after the completion of treatment (week 4), and at the follow-up period (week 12, week 24, week 36, and week 48). The primary outcome in this study is the change in the ACSA between baseline (week 0) and the completion of treatment (week 4).

#### Secondary outcome measures

All the secondary outcomes will be measured at baseline, after the completion of treatment (week 4), and at the follow-up period (week 12, week 24, week 36, and week 48).

A visual analog scale (VAS) will be used to evaluate the craving for ATS [30]. Higher scores reflect a more severe craving for amphetamine.

Hamilton Depression Scale (HAMD-17) and Hamilton Anxiety Scale (HAMA-14) scores [31, 32] will be used to evaluate the severity of depression and anxiety, with higher scores indicating more severe symptoms of depression or anxiety.

Pittsburgh Sleep Quality Index (PSQI) was introduced in 1989 and is a valuable tool for assessing sleep-related problems, which contains 19 items with higher global scores reflecting worse sleep quality [33]. PSQI score will be applied in this study to assess the severity of insomnia.

Montreal Cognitive Assessment (MoCA) Beijing version will be applied to assess the cognitive performance. It contains 30 items, including 5 dimensions (executive function, language, orientation, memory, and abstraction) which is scored ranging from 0 to 30 with a higher score indicating better cognitive performance [34].

Medical Outcomes Study 36-item Short-Form Health Survey (SF-36) is a commonly used instrument for assessing the quality of life [35], which includes the following 8 dimensions: physical functioning, physical role functioning, social role functioning, bodily pain, mental health, emotional role functioning, vitality, and general health perception. SF-36 will be used in this study to assess the overall quality of life of PAAS patients.

### Safety assessment

The occurrence rate of adverse events (AEs), which include broken needles, unbearable needle tingling, local hematoma, infections, and any other discomfort or accidents, will be calculated as (number of cases with AEs/total number of cases)×100%.

The process of intervention will be monitored by a special squad. Participants who develop any adverse events will be managed by licensed clinicians, and all AEs will be collected, recorded, and reported.

### Adherence assessment

The sessions of treatments will be recorded in the CRF to assess the adherence of patients.

### Participant timeline

The participant timeline is described in Table 1.

**Table 1:** Participant timeline

Study period	Baseline		Treatment				Follow-up			
Timepoint (week)	−1	0	1	2	3	4	12	24	36	48
**Participants**										
Screening	√									
Demography	√									
Informed consent	√									
Randomization		√								
Combined medication record	√	√	√	√	√	√				
**Interventions**										
EA group			√	√	√	√				
Waitlist group										
**Outcomes**										
ACSA	√					√	√	√	√	√
VAS	√					√	√	√	√	√
HAMD	√					√	√	√	√	√
HAMA	√					√	√	√	√	√
MoCA	√					√	√	√	√	√
PSQI	√					√	√	√	√	√
SF-36	√					√	√	√	√	√
**Trial safety evaluation**										
Adverse events			√	√	√	√				

*EA group*, electro-acupuncture group; *ACSA*, Amphetamine Cessation Symptom Assessment; *VAS*, visual analog scale; *HAMD*, Hamilton Depression Scale; *HAMA*, Hamilton Anxiety Scale; *MoCA*, Montreal Cognitive Assessment; *PSQI*, Pittsburgh Sleep Quality Index; *SF-36*, Medical Outcomes Study 36-item Short-Form Health Survey

### Quality control and trial monitoring

Regular monitoring will be performed by the trial team according to Good Clinical Practice (GCP) and Clinical Trial Unit standard operating procedures. Following written standard operating procedures, the monitors will verify that the clinical trial is conducted and data are generated, documented, and reported in compliance with the protocol, GCP, and the applicable regulatory requirements.

A trial steering committee (TSC) and data monitoring committee (DMC) will be convened and review trial progress every 6 months. The TSC will provide overall supervision of the trial and ensure its conduct is in accordance with the principles of GCP and the relevant regulations. The TSC will agree with the trial protocol and provide advice to the investigators on all aspects of the trial. The TSC will include members who are independent of the investigators, in particular an independent chairperson. The independent DMC will inform the TSC regarding the accruing trial and safety data, to ensure trial site staff and participants are aware of any relevant safety information and to advise the TSC regarding the appropriateness of continuation of the trial.

### Sample size

A total of 300 participants are expected to be recruited in this study (150 allocated to receive the EA group, 150 to the waitlist group), allowing for 20% loss to follow-up. Though a sample size of 300 participants is believed to ensure internal validity, a pilot study will be performed if the condition permits, given that there are currently no literatures that reported a change of ACSA scores in PAAS patients receiving acupuncture therapy to support the calculation of sample size.

### Data collection and management

The CRF will be documented by the doctors participating in the study, and in each selected case, it must be completed. After the completed CRF is reviewed by the clinical supervisor, the original form will be handed over to the data manager for data entry and management. The trial will set up a database using EpiData2.1 to manage the study data. All electronic data will be secured on a password-protected laptop. The trial statisticians will have access to the data set for the final analysis of trial outcomes. The CRF will be stored in the Acupuncture & Moxibustion Laboratory at Chengdu University of Traditional Chinese Medicine (Chengdu, China). The clinical trial data will be kept for at least 5 years after which it will be destroyed and deleted.

### Measures to improve compliance

Establishing a good relationship between doctors and patients, and providing participants with subsidies for costs.

### Statistical analysis

The statistical analysis will be conducted by independent third-party professional statisticians and will be performed using SAS (Ver9.4). All statistical analyses will follow the principle of intention-to-treat (ITT). All patients who underwent randomization will be included in the final analysis. A two-sided test at a significance level of 95% is deemed as statistically significant.

For the description of baseline features, the mean with the standard deviation or range with the minimum and maximum will be used for continuous data. For homogeneity tests of baseline characteristics between the two groups, a two-sample *t*-test will be used for continuous data. If there are baseline characteristics that exhibit statistically significant differences between the groups, analysis of covariance or logistic regression will be used for analysis and adjustment of the baseline characteristics.

A generalized linear mixed-effects model will be employed to analyze the primary outcome and other repeatedly measured outcomes. Randomization, baseline assessments, stratification factors, assessment times, and time-intervention interaction will be included in the regression models as covariates. It could be used to assess within-participant correlations due to repeated measurements, as well as between-group variations. A *p*-value of less than 0.05 will be considered to indicate statistical significance.

Compliance will be represented primarily by the number of treatment sessions attended. Participants had good compliance, with treatment of more than 80%.

For the comparison of AEs and combined use of drugs between groups, a *χ*^2^ test or Fisher’s exact test will be performed. The data for AEs will be collected from symptoms reported by the patients, and observations by a researcher.

### Discussion

ATS abuse has been increasing yearly all over the world, presenting a serious challenge to global public health [1, 36]. There are multiple reasons that could hinder the treatment of ATS addiction and even contribute to relapse in which PAAS plays a critical role. However, there are few studies focusing on this field resulting in limited treatment strategy and suboptimal clinical outcomes. Recently, acupuncture has been reported by increasing studies to be effective in treating abstinence syndrome, including both the acute withdrawal syndrome and protracted one [17, 37–39]. Therefore, we expect that this study will provide a new approach for the clinical management of ATS withdrawal. We also believe that this study will benefit patients with ATS use disorder by alleviating their protracted symptoms to facilitate their rehabilitation.

However, this study protocol has some limitations. The absence of standard treatment for PAAS makes it difficult to set a positive control in acupuncture studies. Therefore, a waitlist design is selected as the control group based on consideration of ethical consequences, which is also suitable for the pRCT design [25]. On the other hand, the broad inclusion criteria, individualized treatment, complex intervention, and patients’ expectation for acupuncture may cause multiple confounding factors to affect the results [40, 41], which cannot be totally processed through a large sample size or the regression model. Therefore, follow-up studies are needed and will be performed to further explore the potential of acupuncture therapy in this field. If it is validated to be effective in treating PAAS, it can be applied as a complementary and alternative therapy for patients and clinicians to choose.

### Trial status

The research will start in June 2021 and is expected to end in December 2023.

## Data Availability

The data set used and/or analyzed after completion of this research will be provided by the corresponding author upon reasonable request.

## Abbreviations

ATS: Amphetamine-type stimulants
pRCT: Pragmatic randomized controlled trial
PAAS: Protracted amphetamine abstinence syndrome
ACSA: Amphetamine cessation symptom assessment
VAS: Visual analog scale
HAMD: Hamilton Depression Scale
HAMA: Hamilton Anxiety Scale
MoCA: Montreal Cognitive Assessment
PSQI: Pittsburgh Sleep Quality Index
SF-36: Medical Outcomes Study 36-item Short-Form Health Survey
MDMA: Methylenedioxymethamphetamine
TCM: Traditional Chinese medicine
EA: Electro-acupuncture
AEs: Adverse events
CRF: Case report form
